# Protein synthesis inhibition enhances paraptotic death induced by inhibition of cyclophilins in glioblastoma cells

**Published:** 2017-10-02

**Authors:** Lin Wang, Justin H Gundelach, Richard J Bram

**Affiliations:** 1 Department of Biochemistry and Molecular Biology, Mayo Clinic College of Medicine, Rochester, MN, 55905, USA; 2 Department of Immunology, Mayo Clinic College of Medicine, Rochester, MN, 55905, USA; 3 Department of Pediatric and Adolescent Medicine, Mayo Clinic College of Medicine, Rochester, MN, 55905, USA

## Abstract

Treatment of cancer is frequently unsuccessful related to the loss of apoptotic signaling in malignant cells. This is a particular problem for high-grade gliomas, such as Glioblastoma Multiforme (GBM), which are almost universally fatal within a year or so of diagnosis. Novel therapies that capitalize on non-apoptotic cell death pathways may yield more effective outcomes, if their underlying mechanisms can be more completely deciphered. In a recent publication (ref 10), the mechanisms by which cellular cyclophilins support GBM cell survival have been identified. Inhibition of cyclophilins activated paraptosis, which relied on a combination of endoplasmic reticulum (ER) stress and transient activation of autophagy. An important aspect of this effect was the relative rates of cap-dependent versus cap-independent protein synthesis, which were differentially modulated by protein synthesis inhibitors or mTOR inhibition. Although cycloheximide has previously been characterized as an inhibitor of paraptosis, in the case of cyclophilin inhibition, it appears to significantly enhance stress-related paraptosis and cell death. This work reveals an important role for cap-independent protein translation and autophagy in the ability of GBM cells to resist non-apoptotic death, and adds to our understanding of the events that underlie paraptosis.

## Introduction

Glioblastoma Multiforme (GBM) is one of the most frequent brain tumors in adults ^[1]^. Due to its high malignancy, traditional treatments including surgical removal, chemotherapy or radiotherapy do not currently lead to cures ^[2]^. Since the term “apoptosis” was first coined in 1972 ^[3]^, an ever-growing field of interest in cancer therapy has been associated with pharmacologically stimulating apoptotic death in tumor cells. However, frequent gene alterations in cancer cells give rise to resistance to apoptosis, which results in the eventual failure of certain chemotherapeutic drugs ^[4, 5]^. Hence, deploying alternative cell death pathways to eradicate tumor cells could lead to new breakthroughs in cancer treatment. Cyclophilins are a group of chaperones that function as peptidyl-prolyl isomerases (PPIases), whose main function is to catalyze the conversion of proline from cis to trans isomers ^[6]^, in many subcellular compartments. Allowing proline conformational changes is thought to be critically important for proper protein folding to occur ^[7]^. Increased expression of cyclophilins has been found to support viability of many different types of cancer cells ^[8]^, while normal cells seem to be relatively independent of cyclophilins. Previous studies from our laboratory illustrated that cyclophilin B is overexpressed in many cases of GBM, and that genetic depletion of cyclophilin B lead to cell death and elevated ER stress ^[9]^. However, the death mechanism induced by cyclophilin inhibition in GBM cells remains elusive.

In our recently published study, we applied the small molecule cyclophilin inhibitor NIM811 to GBM cells and elucidated a novel non-apoptotic cell death mechanism, which is dependent on inhibition of cyclophilins ^[10]^. We found that NIM811 treated GBM cells established a distinct cell morphology featuring the formation of huge cytoplasmic vacuoles surrounding an intact nucleus. Vacuoles were determined to be of endoplasmic reticulum (ER) origin, which fit the definition of paraptotic cell death ^[11, 12]^. *In vivo* experimentation further confirmed the potency of NIM811 in restricting tumor growth. To obtain a better understanding of cellular pathways that were perturbed by NIM811, we performed RNA-seq to compare the gene status of drug treated cells with controls. Importantly, genes that are responsible for maintaining ER homeostasis were greatly upregulated following NIM811 treatment. Moreover, autophagy related genes were also increased significantly. Western blotting at various time points revealed that the unfolded protein response (UPR) and autophagy were transiently activated but became compromised during prolonged NIM811 incubation. Since NIM811 did not impair proteasome activity, we utilized a bicistronic reporter plasmid to investigate the protein translation status of NIM811 treated cells. By separately quantitating Cap-dependent and Cap-independent translation, we found that the compound increased both types of protein synthesis after 48hrs of exposure, thus contributing to the protein burden in the ER. Remarkably, a brief pre-treatment with the autophagy activators rapamycin or torin-2 only caused significant down-regulation of Cap-dependent translation. In this context, UPR-favored Cap-independent translation was spared. Rapamycin and torin-2 not only reduced ER vacuolization caused by NIM811, but also provided cells with a substantial long-term survival benefit based on colony forming assays. On the contrary, cycloheximide, which has typically been considered a paraptosis inhibitor ^[12–14]^ only transiently delayed vacuoles formation but actually elicited dramatically more cell death following prolonged NIM811 treatment. Finally, shRNA mediated depletion of cyclophilin A or B in GBM cells cause them to become more sensitive to NIM811, thus reinforcing that NIM811 induced paraptosis was cyclophilin-dependent.

## Conclusion

We propose that NIM811 triggers a series of cellular events, starting with the generation of misfolded or unfolded protein in ER. Initially, the cellular response was to activate the UPR and autophagy to facilitate unfolded protein clearance. Within a short time period, however, these two mechanisms become persistently inactivated and protein aggregates begin to accumulate in the ER. To minimize cellular damage, the ER appears to form compartments to isolate these improperly folded proteins, and this process is observed as cellular vacuolization. Since NIM811 fosters escalating protein translation, ER stress concordantly increases eventually leading to cell death. Cycloheximide unselectively suppresses both Cap-dependent and Cap-independent translation, and also inhibits pro-survival autophagy. Therefore, brief pre-treatment with cycloheximide exacerbated NIM811 induced paraptosis.

These findings expand our understanding of paraptoticcell death and allow us to identify crucial cellular mechanisms that determine the fate of cells on whether to live or to die. Although in GBM cells, autophagy is not greatly enhanced above normal cells, it remains indispensible for cancer cells to circumvent ER stress associated paraptosis. These studies reiterate the importance of cyclophilins in aiding cancer cell survival under stress conditions, and support their potential as therapeutic targets.
